# Crystal structure of 5-*O*-benzoyl-2,3-*O*-iso­propyl­idene-d-ribono-1,4-lactone

**DOI:** 10.1107/S2056989017002043

**Published:** 2017-02-17

**Authors:** Adailton J. Bortoluzzi, Gustavo P. Silveira, Marcus M. Sá

**Affiliations:** a Depto. de Química - Universidade Federal de Santa Catarina, 88040-900 – Florianópolis, Santa Catarina, Brazil; b Departamento de Química Orgânica - Instituto de Química, Universidade Federal do Rio Grande do Sul, 91501-970 – Porto Alegre, Rio Grande do Sul, Brazil

**Keywords:** crystal structure, absolute configuration, d-ribono-1,4-lactone, fused five-membered ring system

## Abstract

In the title compound, C_15_H_16_O_6_, obtained from the acyl­ation reaction between 2,3-*O*-iso­propyl­idene-d-ribono-1,4-lactone and benzoyl chloride, the known absolute configuration for the lactone moiety of the ester substituent has been confirmed. The five-membered rings of the bicyclic lactone–dioxolane moiety both show envelope conformations and form a dihedral angle of 19.82 (7)° between the lactone ring and the benzene ring. In the crystal, mol­ecules of the acyl­ated sugar are linked by very weak inter­molecular C—H⋯O inter­actions, forming a three-dimensional network.

## Abstract 2

In the title compound, obtained from the acyl­ation reaction between 2,3-*O*-iso­propyl­idene-d-ribono-1,4-lactone and benzoyl chloride, the known absolute configuration for the lactone moiety of the ester substituent has been confirmed. The five-membered rings of the bicyclic lactone–dioxolane moiety both show envelope conformations and form a dihedral angle of 19.82 (7)° between the lactone ring and the benzene ring.

## Chemical context   

Aldonolactones are modified sugars with the anomeric center in its higher oxidation state. They have been widely employed as versatile chiral pools for the synthesis of biologically important mol­ecules due to their abundance from sustainable resources as well as their low cost (Corma *et al.*, 2007 ▸; Han *et al.*, 1993 ▸; Silveira *et al.*, 2015 ▸). However, the chemical complexity associated with most carbohydrates, which is mainly due to the subtle differences in the reactivity of similar hydroxyl groups and the simultaneous existence of tautomeric species in equilibrium, may lead to unexpected transformations such as rearrangements and functional group migrations (Baggett *et al.*, 1985 ▸; Sá *et al.*, 2008 ▸). Therefore, the synthesis of new carbohydrate-based mol­ecules often relies on single crystal X-ray analysis for correct structural and conformational assignments (Booth *et al.*, 2009 ▸; Czugler & Pintér, 2011 ▸; Sales & Silveira, 2015 ▸). In a continuation of our research on the chemistry of carbohydrates (Bortoluzzi *et al.*, 2011 ▸; Cardoso *et al.*, 2015 ▸; Sá *et al.*, 2002 ▸, 2008 ▸; Sebrão *et al.*, 2011 ▸), we describe herein the crystal structure of 5-*O*-benzoyl-2,3-*O*-iso­propyl­idene-d-ribono-1,4-lactone, C_15_H_16_O_6_, (I).
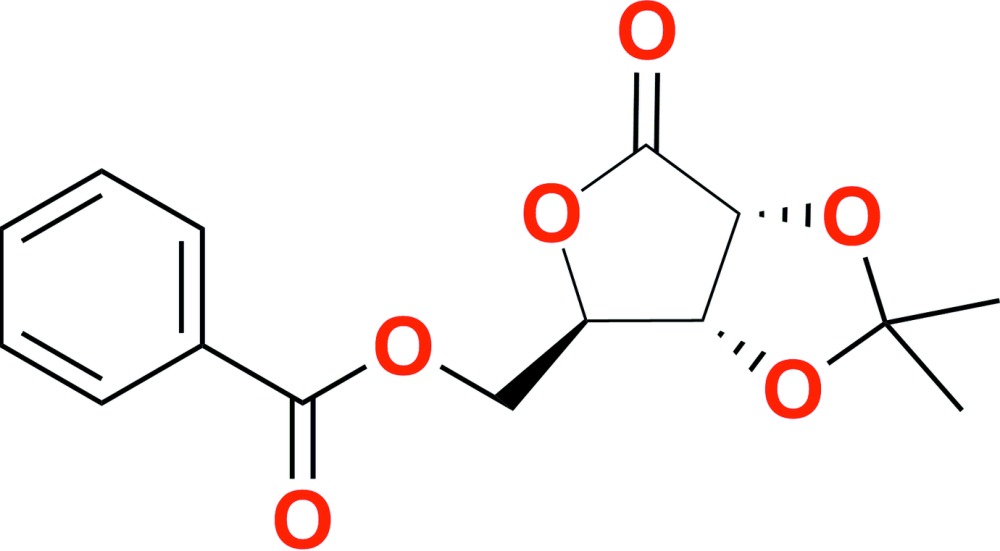


## Structural commentary   

**Figure 1:**
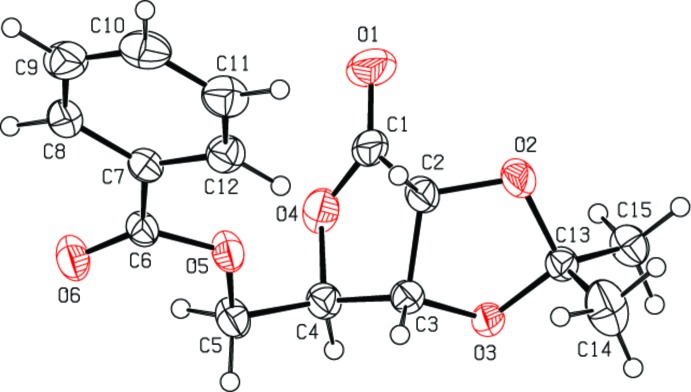
The mol­ecular structure of (I), with the atom-labeling scheme. Displacement ellipsoids are shown at the 40% probability level.

Compound (I) (Fig. 1 ▸) has three chiral centers with the absolute configuration determined as C2(*R*),C3(*S*),C4(*R*) [Flack factor 0.05 (3) for 1078 quotients (Parsons *et al.*, 2013 ▸)], which is consistent with the known configuration for the lactone ring (Sá *et al.*, 2008 ▸; Sales & Silveira, 2015 ▸). Both five-membered rings of the bicyclic lactone-dioxolane moiety show envelope conformations. However, the dioxolane ring adopts a more regular envelope conformation, comparing the puckering parameters for O3 [*Q*(2) = 0.3141 (15) Å, φ(2) = 284.5 (3)°] with those for C3 [*Q*(2) = 0.2261 (17) Å, φ(2) = 121.9 (4)°], but this ring is slightly twisted about the C1—C2 bond. This is indicated by the comparative torsion angles C13—O2—C2—C3 for the dioxolane ring and C4—O4—C1—C2 of the lactone ring of 1.55 (18) and 6.87 (16)°, respectively. The dihedral angle between the mean plane of the benzene ring and that of the ester group (O6/C6/O5/C5) is 16.59 (9)°. All bond lengths and angles observed for (I) are within the expected range for organic compounds (Bruno *et al.*, 2004 ▸).

## Supra­molecular features   

**Figure 2:**
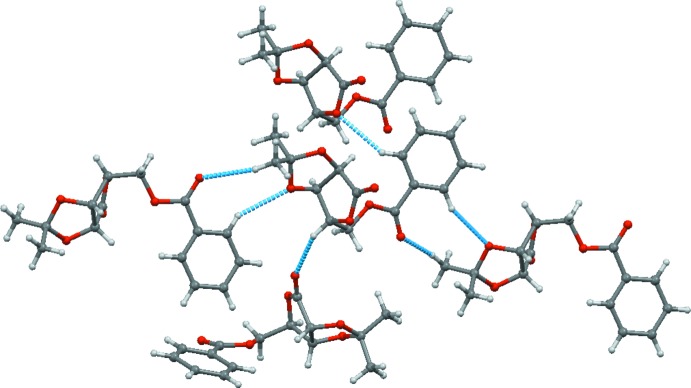
Weak C—H⋯O contacts around the independent mol­ecule.

The mol­ecules of (I) are stacked along the crystallographic *a* axis. Several weak C—H⋯O inter­actions (Table 1 ▸, Fig. 2 ▸) are observed in the crystal, forming an intricate three-dimensional network.

## Database survey   

A search in the current version of the Cambridge Structural Database (Version 5.37, November 2016; Groom *et al.*, 2016 ▸) for structures containing a bicyclic lactone-dioxolane moiety revealed only seven entries (refcodes: JOBJOZ, OCAVOE, VAXCAA, VENBAS, YISHAJ, YISHAK01 and YISHOX), which are related to articles published from 1991 to 2012.

## Synthesis and crystallization   

**Figure 3:**
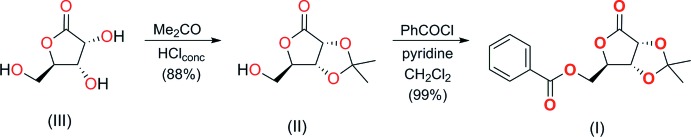
Reaction scheme for the synthesis of compound (I).

5-*O*-Benzoyl-2,3-*O*-iso­propyl­idene-d-ribono-1,4-lactone (I) was prepared in qu­anti­tative yield through the acyl­ation of 2,3-*O*-iso­propyl­idene-d-ribono-1,4-lactone (II) with benzoyl chloride in pyridine followed by aqueous work-up and purif­ication according to the reported method (Sá *et al.*, 2002 ▸). The two-step preparation of (I) is shown in the reaction scheme (Fig. 3 ▸). Slow crystallization from ethanol solution furnished single crystals (m.p. 371–372 K), allowing structural elucidation by X-ray crystallographic techniques. The absolute configuration for (I) was established by refinement of the Flack parameter and is in complete agreement with previous assignments made on the basis of hydrogen- and carbon-NMR shifts for the starting d-ribono-1,4-lactones (II) and (III), and on the homogeneity of the reaction product.

## Refinement   

Crystal data, data collection and structure refinement details are summarized in Table 2 ▸. H atoms were placed in idealized positions and allowed to ride with C—H distances of 0.95 Å (CH_Ar_), 1.00 Å (CH), 0.99 Å (CH_2_) or 0.98 Å (CH_3_) with *U*_iso_(H) = 1.2*U*_eq_(C) or 1.5*U*_eq_(C_meth­yl_).

## Supplementary Material

Crystal structure: contains datablock(s) I, Global. DOI: 10.1107/S2056989017002043/zs2372sup1.cif

Structure factors: contains datablock(s) I. DOI: 10.1107/S2056989017002043/zs2372Isup2.hkl

Supporting information file. DOI: 10.1107/S2056989017002043/zs2372Isup3.mol

Supporting information file. DOI: 10.1107/S2056989017002043/zs2372Isup4.cml

CCDC reference: 1531628

Additional supporting information:  crystallographic information; 3D view; checkCIF report

## Figures and Tables

**Table 1:** Hydrogen-bond geometry (Å, °)

*D*—H⋯*A*	*D*—H	H⋯*A*	*D*⋯*A*	*D*—H⋯*A*
C4—H4⋯O1^i^	1.00	2.52	3.2381 (19)	128
C8—H8⋯O3^ii^	0.95	2.65	3.4951 (19)	148
C12—H12⋯O4^iii^	0.95	2.66	3.4682 (19)	143
C15—H15*A*⋯O6^iv^	0.98	2.59	3.551 (2)	166

Symmetry codes: (i) 
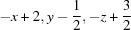
; (ii) 
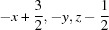
; (iii) 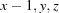; (iv) 
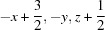
.

**Table 2:** Experimental details

Crystal data	
Chemical formula	C_15_H_16_O_6_
*M*_r_	292.28
Crystal system, space group	Orthorhombic, *P*2_1_2_1_2_1_
Temperature (K)	200
*a*, *b*, *c* (Å)	5.7574 (1), 12.5703 (3), 20.1888 (4)
*V* (Å^3^)	1461.11 (5)
*Z*	4
Radiation type	Cu *K*α
μ (mm^−1^)	0.87
Crystal size (mm)	0.20 × 0.18 × 0.16

Data collection	
Diffractometer	Bruker APEXII CCD
Absorption correction	Multi-scan (*SADABS*; Bruker, 2009 ▸)
*T*_min_, *T*_max_	0.682, 0.753
No. of measured, independent and observed [*I* > 2σ(*I*)] reflections	12424, 2655, 2635
*R*_int_	0.024
(sin θ/λ)_max_ (Å^−1^)	0.602

Refinement	
*R*[*F* ^2^ > 2σ(*F* ^2^)], *wR*(*F* ^2^), *S*	0.024, 0.068, 1.02
No. of reflections	2655
No. of parameters	193
H-atom treatment	H-atom parameters constrained
Δρ_max_, Δρ_min_ (e Å^−3^)	0.13, −0.10
Absolute structure	Flack *x* determined using 1078 quotients [(*I* ^+^)−(*I* ^−^)]/[(*I* ^+^)+(*I* ^−^)] (Parsons *et al.*, 2013 ▸)
Absolute structure parameter	0.05 (3)

Computer programs: *APEX2* and *SAINT* (Bruker, 2009 ▸), *SHELXS97* (Sheldrick, 2008 ▸), *SHELXL2012* (Sheldrick, 2015 ▸), *PLATON* (Spek, 2009 ▸), *Mercury* (Macrae *et al.*, 2008 ▸) and *publCIF* (Westrip, 2010 ▸).

## supplementary crystallographic information

### Crystal data

**Table:**

C_15_H_16_O_6_	*D*_x_ = 1.329 Mg m^−^^3^
*M**_r_* = 292.28	Melting point = 371–372 K
Orthorhombic, *P*2_1_2_1_2_1_	Cu *K*α radiation, λ = 1.54178 Å
*a* = 5.7574 (1) Å	Cell parameters from 9982 reflections
*b* = 12.5703 (3) Å	θ = 4.1–68.1°
*c* = 20.1888 (4) Å	µ = 0.87 mm^−^^1^
*V* = 1461.11 (5) Å^3^	*T* = 200 K
*Z* = 4	Irregular block, colourless
*F*(000) = 616	0.20 × 0.18 × 0.16 mm

### Data collection

**Table:**

Bruker APEXII CCD diffractometer	2635 reflections with *I* > 2σ(*I*)
Radiation source: Cu IµS microfocus X-ray source	*R*_int_ = 0.024
φ and ω scans	θ_max_ = 68.1°, θ_min_ = 4.1°
Absorption correction: multi-scan (SADABS; Bruker, 2009)	*h* = −5→6
*T*_min_ = 0.682, *T*_max_ = 0.753	*k* = −14→14
12424 measured reflections	*l* = −24→24
2655 independent reflections	

### Refinement

**Table:**

Refinement on *F*^2^	H-atom parameters constrained
Least-squares matrix: full	*w* = 1/[σ^2^(*F*_o_^2^) + (0.044*P*)^2^ + 0.1285*P*] where *P* = (*F*_o_^2^ + 2*F*_c_^2^)/3
*R*[*F*^2^ > 2σ(*F*^2^)] = 0.024	(Δ/σ)_max_ < 0.001
*wR*(*F*^2^) = 0.068	Δρ_max_ = 0.13 e Å^−^^3^
*S* = 1.02	Δρ_min_ = −0.10 e Å^−^^3^
2655 reflections	Extinction correction: SHELXL2012 (Sheldrick, 2015), Fc^*^=kFc[1+0.001xFc^2^λ^3^/sin(2θ)]^-1/4^
193 parameters	Extinction coefficient: 0.0059 (7)
0 restraints	Absolute structure: Flack *x* determined using 1078 quotients [(*I*^+^)-(*I*^-^)]/[(*I*^+^)+(*I*^-^)] (Parsons *et al.*, 2013)
Hydrogen site location: inferred from neighbouring sites	Absolute structure parameter: 0.05 (3)

### Special details

**Table:**

Geometry. All esds (except the esd in the dihedral angle between two l.s. planes) are estimated using the full covariance matrix. The cell esds are taken into account individually in the estimation of esds in distances, angles and torsion angles; correlations between esds in cell parameters are only used when they are defined by crystal symmetry. An approximate (isotropic) treatment of cell esds is used for estimating esds involving l.s. planes.

### Fractional atomic coordinates and isotropic or equivalent isotropic displacement parameters (Å^2^)

**Table:**

	*x*	*y*	*z*	*U*_iso_*/*U*_eq_	
O5	0.67330 (19)	0.01217 (8)	0.65402 (5)	0.0398 (3)	
O3	0.72276 (19)	0.09129 (8)	0.85566 (5)	0.0350 (3)	
O4	1.0094 (2)	0.11588 (10)	0.73280 (5)	0.0461 (3)	
O1	0.9394 (4)	0.28572 (11)	0.70948 (7)	0.0756 (5)	
O2	0.5870 (3)	0.24630 (9)	0.81330 (6)	0.0514 (3)	
O6	0.8024 (2)	−0.07705 (9)	0.56539 (6)	0.0476 (3)	
C10	0.1551 (3)	0.15260 (14)	0.47990 (11)	0.0540 (4)	
H10	0.0393	0.1887	0.4550	0.065*	
C9	0.3372 (4)	0.10297 (15)	0.44780 (9)	0.0537 (5)	
H9	0.3466	0.1057	0.4009	0.064*	
C8	0.5059 (3)	0.04941 (12)	0.48336 (8)	0.0433 (4)	
H8	0.6298	0.0146	0.4611	0.052*	
C7	0.4921 (3)	0.04704 (11)	0.55213 (7)	0.0344 (3)	
C6	0.6721 (3)	−0.01296 (11)	0.58914 (7)	0.0338 (3)	
C5	0.8325 (3)	−0.04652 (12)	0.69549 (8)	0.0408 (4)	
H5A	0.9779	−0.0620	0.6712	0.049*	
H5B	0.7620	−0.1147	0.7096	0.049*	
C4	0.8814 (3)	0.02301 (12)	0.75469 (7)	0.0359 (3)	
H4	0.9769	−0.0175	0.7875	0.043*	
C3	0.6671 (3)	0.06717 (11)	0.78883 (7)	0.0325 (3)	
H3	0.5274	0.0205	0.7842	0.039*	
C13	0.5968 (3)	0.18470 (12)	0.87332 (7)	0.0321 (3)	
C14	0.3543 (3)	0.15824 (18)	0.89603 (12)	0.0622 (6)	
H14A	0.2719	0.1206	0.8606	0.093*	
H14C	0.2713	0.2241	0.9068	0.093*	
H14B	0.3621	0.1129	0.9355	0.093*	
C1	0.8711 (4)	0.20229 (13)	0.73049 (7)	0.0451 (4)	
C2	0.6335 (3)	0.17742 (12)	0.75949 (7)	0.0384 (4)	
H2	0.5079	0.1791	0.7253	0.046*	
C11	0.1417 (3)	0.14971 (15)	0.54823 (10)	0.0509 (4)	
H11	0.0162	0.1837	0.5703	0.061*	
C12	0.3101 (3)	0.09759 (13)	0.58439 (8)	0.0405 (3)	
H12	0.3016	0.0963	0.6314	0.049*	
C15	0.7316 (3)	0.24526 (13)	0.92440 (8)	0.0419 (4)	
H15A	0.7527	0.2008	0.9638	0.063*	
H15B	0.6464	0.3099	0.9365	0.063*	
H15C	0.8837	0.2647	0.9063	0.063*	

### Atomic displacement parameters (Å^2^)

**Table:**

	*U*^11^	*U*^22^	*U*^33^	*U*^12^	*U*^13^	*U*^23^
O5	0.0462 (6)	0.0393 (5)	0.0339 (5)	0.0110 (5)	−0.0058 (4)	−0.0088 (4)
O3	0.0450 (5)	0.0325 (5)	0.0275 (5)	0.0074 (4)	0.0000 (4)	0.0018 (4)
O4	0.0451 (6)	0.0534 (7)	0.0399 (6)	−0.0117 (5)	0.0069 (5)	−0.0091 (5)
O1	0.1282 (15)	0.0489 (7)	0.0498 (7)	−0.0368 (9)	0.0041 (9)	0.0046 (6)
O2	0.0832 (9)	0.0372 (6)	0.0340 (5)	0.0233 (6)	−0.0032 (6)	−0.0011 (5)
O6	0.0554 (7)	0.0464 (6)	0.0410 (6)	0.0153 (5)	−0.0015 (5)	−0.0115 (5)
C10	0.0549 (10)	0.0435 (9)	0.0636 (11)	0.0005 (8)	−0.0154 (9)	0.0109 (8)
C9	0.0779 (13)	0.0430 (9)	0.0402 (8)	−0.0036 (9)	−0.0121 (9)	0.0041 (7)
C8	0.0576 (10)	0.0350 (8)	0.0374 (8)	0.0003 (7)	0.0002 (7)	−0.0051 (6)
C7	0.0386 (8)	0.0280 (7)	0.0365 (7)	−0.0043 (6)	−0.0002 (6)	−0.0029 (5)
C6	0.0385 (7)	0.0286 (7)	0.0343 (7)	−0.0032 (6)	0.0010 (6)	−0.0061 (5)
C5	0.0468 (9)	0.0370 (7)	0.0386 (8)	0.0107 (7)	−0.0064 (7)	−0.0072 (6)
C4	0.0393 (7)	0.0345 (7)	0.0338 (7)	0.0038 (6)	−0.0019 (6)	−0.0011 (6)
C3	0.0359 (7)	0.0308 (7)	0.0307 (7)	0.0005 (6)	−0.0015 (5)	0.0001 (5)
C13	0.0336 (7)	0.0314 (7)	0.0312 (6)	0.0042 (6)	0.0006 (6)	−0.0006 (5)
C14	0.0376 (9)	0.0652 (12)	0.0839 (14)	−0.0097 (9)	0.0146 (9)	−0.0254 (11)
C1	0.0713 (11)	0.0384 (8)	0.0257 (7)	−0.0142 (8)	−0.0022 (7)	−0.0031 (6)
C2	0.0534 (9)	0.0326 (7)	0.0291 (6)	0.0077 (7)	−0.0082 (7)	−0.0027 (6)
C11	0.0408 (9)	0.0483 (9)	0.0636 (11)	0.0046 (8)	0.0013 (8)	0.0053 (8)
C12	0.0375 (7)	0.0409 (8)	0.0430 (8)	−0.0017 (7)	0.0034 (7)	0.0017 (6)
C15	0.0400 (8)	0.0411 (8)	0.0445 (8)	0.0018 (7)	−0.0064 (7)	−0.0053 (7)

### Geometric parameters (Å, º)

**Table:**

O5—C6	1.3475 (17)	C5—H5A	0.9900
O5—C5	1.4440 (18)	C5—H5B	0.9900
O3—C3	1.4196 (17)	C4—C3	1.518 (2)
O3—C13	1.4253 (17)	C4—H4	1.0000
O4—C1	1.348 (2)	C3—C2	1.520 (2)
O4—C4	1.4496 (19)	C3—H3	1.0000
O1—C1	1.198 (2)	C13—C15	1.498 (2)
O2—C2	1.4148 (18)	C13—C14	1.507 (2)
O2—C13	1.4391 (17)	C14—H14A	0.9800
O6—C6	1.2008 (18)	C14—H14C	0.9800
C10—C9	1.381 (3)	C14—H14B	0.9800
C10—C11	1.382 (3)	C1—C2	1.520 (3)
C10—H10	0.9500	C2—H2	1.0000
C9—C8	1.383 (3)	C11—C12	1.379 (2)
C9—H9	0.9500	C11—H11	0.9500
C8—C7	1.391 (2)	C12—H12	0.9500
C8—H8	0.9500	C15—H15A	0.9800
C7—C12	1.388 (2)	C15—H15B	0.9800
C7—C6	1.484 (2)	C15—H15C	0.9800
C5—C4	1.507 (2)		
			
C6—O5—C5	116.56 (11)	C4—C3—H3	113.4
C3—O3—C13	107.39 (10)	C2—C3—H3	113.4
C1—O4—C4	111.05 (12)	O3—C13—O2	104.63 (11)
C2—O2—C13	108.05 (11)	O3—C13—C15	109.11 (12)
C9—C10—C11	119.93 (17)	O2—C13—C15	109.05 (13)
C9—C10—H10	120.0	O3—C13—C14	111.45 (14)
C11—C10—H10	120.0	O2—C13—C14	109.80 (15)
C8—C9—C10	120.63 (17)	C15—C13—C14	112.49 (14)
C8—C9—H9	119.7	C13—C14—H14A	109.5
C10—C9—H9	119.7	C13—C14—H14C	109.5
C9—C8—C7	119.23 (17)	H14A—C14—H14C	109.5
C9—C8—H8	120.4	C13—C14—H14B	109.5
C7—C8—H8	120.4	H14A—C14—H14B	109.5
C12—C7—C8	120.13 (15)	H14C—C14—H14B	109.5
C12—C7—C6	121.59 (13)	O1—C1—O4	121.6 (2)
C8—C7—C6	118.26 (14)	O1—C1—C2	127.7 (2)
O6—C6—O5	122.84 (13)	O4—C1—C2	110.64 (12)
O6—C6—C7	125.20 (13)	O2—C2—C3	106.43 (11)
O5—C6—C7	111.96 (12)	O2—C2—C1	109.89 (14)
O5—C5—C4	106.38 (12)	C3—C2—C1	102.91 (13)
O5—C5—H5A	110.5	O2—C2—H2	112.4
C4—C5—H5A	110.5	C3—C2—H2	112.4
O5—C5—H5B	110.5	C1—C2—H2	112.4
C4—C5—H5B	110.5	C12—C11—C10	120.07 (18)
H5A—C5—H5B	108.6	C12—C11—H11	120.0
O4—C4—C5	108.68 (12)	C10—C11—H11	120.0
O4—C4—C3	104.91 (11)	C11—C12—C7	120.00 (16)
C5—C4—C3	114.84 (13)	C11—C12—H12	120.0
O4—C4—H4	109.4	C7—C12—H12	120.0
C5—C4—H4	109.4	C13—C15—H15A	109.5
C3—C4—H4	109.4	C13—C15—H15B	109.5
O3—C3—C4	109.02 (12)	H15A—C15—H15B	109.5
O3—C3—C2	101.79 (11)	C13—C15—H15C	109.5
C4—C3—C2	105.05 (12)	H15A—C15—H15C	109.5
O3—C3—H3	113.4	H15B—C15—H15C	109.5
			
C11—C10—C9—C8	0.5 (3)	C3—O3—C13—C15	−150.12 (12)
C10—C9—C8—C7	−0.8 (3)	C3—O3—C13—C14	85.06 (16)
C9—C8—C7—C12	0.3 (2)	C2—O2—C13—O3	18.81 (16)
C9—C8—C7—C6	178.64 (14)	C2—O2—C13—C15	135.43 (14)
C5—O5—C6—O6	−3.3 (2)	C2—O2—C13—C14	−100.90 (16)
C5—O5—C6—C7	176.29 (12)	C4—O4—C1—O1	−175.01 (14)
C12—C7—C6—O6	162.66 (16)	C4—O4—C1—C2	6.87 (16)
C8—C7—C6—O6	−15.7 (2)	C13—O2—C2—C3	1.55 (18)
C12—C7—C6—O5	−16.96 (19)	C13—O2—C2—C1	−109.21 (14)
C8—C7—C6—O5	164.70 (13)	O3—C3—C2—O2	−21.16 (17)
C6—O5—C5—C4	155.62 (13)	C4—C3—C2—O2	−134.80 (13)
C1—O4—C4—C5	104.04 (14)	O3—C3—C2—C1	94.39 (12)
C1—O4—C4—C3	−19.24 (15)	C4—C3—C2—C1	−19.24 (14)
O5—C5—C4—O4	−66.95 (15)	O1—C1—C2—O2	−56.6 (2)
O5—C5—C4—C3	50.16 (17)	O4—C1—C2—O2	121.41 (13)
C13—O3—C3—C4	144.09 (12)	O1—C1—C2—C3	−169.62 (16)
C13—O3—C3—C2	33.44 (14)	O4—C1—C2—C3	8.36 (15)
O4—C4—C3—O3	−84.91 (13)	C9—C10—C11—C12	0.2 (3)
C5—C4—C3—O3	155.85 (12)	C10—C11—C12—C7	−0.7 (3)
O4—C4—C3—C2	23.54 (14)	C8—C7—C12—C11	0.4 (2)
C5—C4—C3—C2	−95.70 (15)	C6—C7—C12—C11	−177.87 (15)
C3—O3—C13—O2	−33.54 (14)		

### Hydrogen-bond geometry (Å, º)

**Table:**

*D*—H···*A*	*D*—H	H···*A*	*D*···*A*	*D*—H···*A*
C4—H4···O1^i^	1.00	2.52	3.2381 (19)	128
C5—H5*B*···O1^i^	0.99	2.68	3.139 (2)	108
C8—H8···O3^ii^	0.95	2.65	3.4951 (19)	148
C12—H12···O4^iii^	0.95	2.66	3.4682 (19)	143
C15—H15*A*···O6^iv^	0.98	2.59	3.551 (2)	166
C15—H15*C*···O6^v^	0.98	2.75	3.497 (2)	134

Symmetry codes: (i) −*x*+2, *y*−1/2, −*z*+3/2; (ii) −*x*+3/2, −*y*, *z*−1/2; (iii) *x*−1, *y*, *z*; (iv) −*x*+3/2, −*y*, *z*+1/2; (v) −*x*+2, *y*+1/2, −*z*+3/2.
